# Surface Morphology Transformation Under High-Temperature Annealing of Ge Layers Deposited on Si(100)

**DOI:** 10.1186/s11671-016-1588-1

**Published:** 2016-08-19

**Authors:** A. A. Shklyaev, A. V. Latyshev

**Affiliations:** 1 A.V. Rzhanov Institute of Semiconductor Physics, SB RAS, Novosibirsk, 630090 Russia; 2 Novosibirsk State University, Novosibirsk, 630090 Russia

**Keywords:** Ge layers on Si(100), Strain-induced melting, Porous SiGe layers, Dislocation-rich SiGe layers, Dewetting, 64.75.St, 68.55.ag, 68.37.Ff, 68.37.Hk

## Abstract

We study the surface morphology and chemical composition of SiGe layers after their formation under high-temperature annealing at 800–1100 °C of 30–150 nm Ge layers deposited on Si(100) at 400–500 °C. It is found that the annealing leads to the appearance of the SiGe layers of two types, i.e., porous and continuous. The continuous layers have a smoothened surface morphology and a high concentration of threading dislocations. The porous and continuous layers can coexist. Their formation conditions and the ratio between their areas on the surface depend on the thickness of deposited Ge layers, as well as on the temperature and the annealing time. The data obtained suggest that the porous SiGe layers are formed due to melting of the strained Ge layers and their solidification in the conditions of SiGe dewetting on Si. The porous and dislocation-rich SiGe layers may have properties interesting for applications.

## Background

Different semiconductor heterostructures made of Si and Ge are the basic materials for the fabrication of commercial circuits for microelectronics. The use of Ge layers on Si significantly improves their performance due to greater charge carrier mobility in Ge. At the same time, Ge layers on Si are studied in order to create light sources and detectors for the near infrared spectrum range, as well as solar cells and Ge lasers [1–3]. The Ge growth on Si usually occurs through the Stranski-Krastanov growth mode, leading to three-dimensional (3D) island formation [4–6]. The main disadvantage of Si/Ge heterostructures consists in their inhomogeneity in lattice strain and chemical composition caused by Si and Ge lattice constant mismatch. Such structures are characterized by a wide electronic states distribution that is an obstacle for the efficient devices fabrication in some cases. Possible approaches to obtain the homogeneity in the Si/Ge structures are the use of lower [7, 8] or higher temperatures [9] than those usually applied for their preparation.

Ge depositions on Si(100) at temperatures up to 500 °C leads to the formation of 3D islands having different shapes and sizes depending on the substrate temperature and on the amount and deposition rate of Ge [4, 7, 10, 11]. The island formation occurs by means of the processes such as surface diffusion and incorporation of deposited adatoms into energetically preferable sites on the surface. At temperatures of 500–750 °C, the Si-Ge intermixing in surface layers appears to be an additional process governing the composition and strain distribution in the growing structures [12–15]. When the temperature is above 800 °C, deposited Ge atom diffusion in the Si substrate becomes significant. If the Ge deposition rate is not high enough, all deposited Ge atoms diffuse into the substrate and the island growth does not occur [9].

The Ge and SiGe islands grown on Si(100) undergo a strong strain due to the lattice mismatch between Si and Ge and, therefore, they are thermally unstable. The strain reduction can be achieved by the formation of more homogeneous distribution of chemical composition by means of Si-Ge intermixing under high-temperature annealing. Annealing can also produce significant changes in the surface morphology [16]. The temperature effects were studied with respect to compositional atomic ordering and surface morphology evolution for the SiGe islands prepared by the deposition of relatively low Ge coverages (up to several nm) [17, 18]. In the first part of this work, we study the surface morphology obtained by the deposition of relatively high Ge coverages (30–150 nm) on Si(100) at temperatures of 400–500 °C. The obtained images of surface morphology show that, as the Ge coverage increases, the islands merge in ridges. The effects of high-temperature annealing were studied then in order to reveal the changes in the surface morphology and in the chemical composition of the grown structures. Annealing at 800–900 °C led to the formation of two different surface areas. One of them is composed of open Si substrate windows and relatively large ridges around them. The formation of such porous SiGe layers looks like the result of strain-facilitated melting with subsequent solidification in the conditions of SiGe dewetting on Si(100). The other surface area is the continuous SiGe layer which is formed by smoothing of the grown islands or ridges with the material transfer in the areas between them. Such surface morphology transformation is accompanied by the appearance of a high threading dislocation concentration. The areas with two essentially different surface morphologies can coexist on the surface. The ratio between sizes of these two areas depends on the temperature and the annealing time, and it is determined by the competition between processes such as strain-facilitated melting from one side and a gradual mass transport along the surface and Si-Ge intermixing with Ge diffusion into the substrate from the other side.

## Methods

The growth experiments were carried out in an ultrahigh-vacuum chamber with a base pressure of about 1 × 10^−10^ Torr. The chamber was equipped with a scanning tunneling microscope (STM) manufactured by Omicron. A 10 × 2 × 0.3 mm^3^ sample was cut from an n-type Si(100) wafer with a miscut angle of <10´ and a resistivity of 5–20 Ω cm. Clean Si surfaces were prepared by flash direct-current heating at 1250–1300 °C. A Knudsen cell with a BN crucible was used for Ge deposition at the rate up to 1.0 nm/min, which was calibrated with the STM for the initial stage of Ge growth on the Si(111) surface at the low temperature of 450 °C. The Ge growth on Si(100) surfaces was carried out at the temperatures of 400 and 500 °C. The post-growth sample annealing was performed at temperatures from 800 to 1100 °C for various durations up to 3 h. The sample temperature was measured using an IMPAC IGA 12 pyrometer. After the removal of the samples from the growth chamber, their scanning electron microscope (SEM) images were obtained using a Pioneer microscope manufactured by Raith. The chemical composition of the grown layers was measured using energy-dispersive X-ray spectroscopy (EDX) of SEM SU8220 made by Hitachi. To obtain a better spatial resolution in measuring the chemical composition along the certain line, the incident e-beam energy was reduced to 3 keV. The EDX for such low e-beam energy was calibrated using samples with known SiGe compositions.

After electrochemical etching, the W STM tips were sharpened by cutting the tip apexes from several sides using the 30 kV focused Ga ion beam of a 1540 XB Carl Zeiss microscope. As a result, the typical opening angle of the STM tip apexes, estimated from the STM images, was about 20°. The STM images of the surfaces covered with Ge were usually obtained with the STM tip bias voltage of −2.0 V and the constant current of 10 pA. The STM image processing with free software Gwyddion was used for the generation of bird view images of the surface morphology and height profiles.

## Results and Discussion

**Fig. 1:**
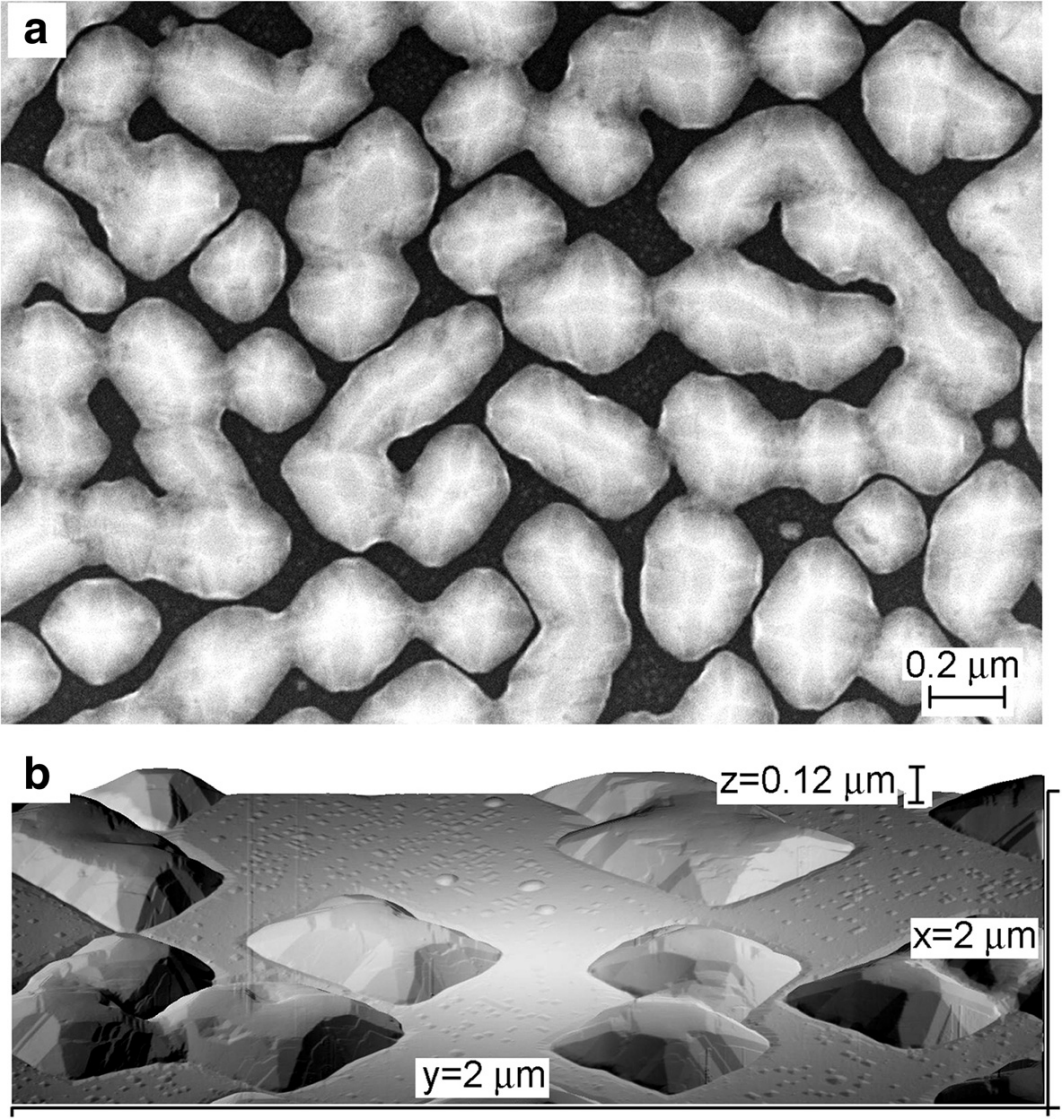
**a** SEM and **b** STM images of the surfaces obtained by **a** 30 and **b** 20 nm Ge depositions on Si(100) at 400 and 500 °C, respectively

Ge growth on Si(100) at 400–500 °C proceeds through the formation of hut clusters and square-based pyramidal islands [4]. Some of them develop then into the large dome-like islands [19]. For relatively large Ge coverages and at the same Ge deposition rate, the concentration of dome-like islands decreases, as the growth temperature increases, as shown for 400 and 500 °C in Fig. 1, indicating the typical temperature dependence of the island concentration [20]. When the deposited Ge coverage reaches 30 nm, the distance between the dome-like islands becomes so small that some of the islands coalesce. Interesting is that the hut clusters and square-base pyramidal islands remain on the surface areas between growing dome-like islands [Fig. 1(b)]. At the growth temperature of 500 °C, the trenches appeared around the islands (Fig. 1b), indicating the transfer of Si from the substrate into the islands [10, 13, 15, 21].

**Fig. 2:**
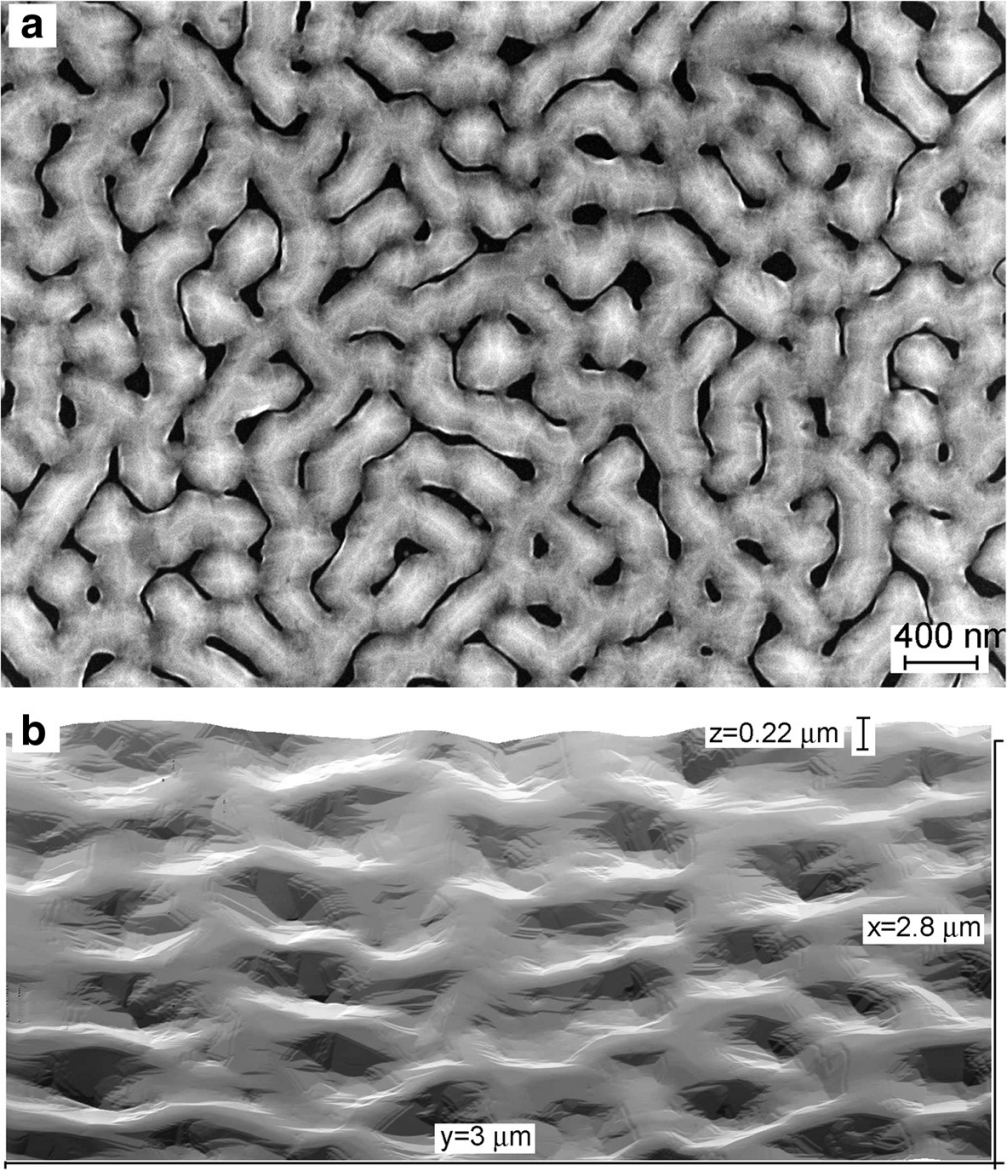
**a** SEM and **b** STM images of the surfaces obtained by **a** 60 and **b** 150 nm Ge depositions on Si(100) at 400 °C

When the Ge coverage exceeded 30 nm, the islands formed continuous ridges (Fig. 2a). The use of 400 °C provides the formation of a more homogeneous surface morphology than that formed by Ge depositions at 500 °C. The Si(100) surface was completely covered with Ge islands, and the flat surface areas were not observed between them when the Ge coverage deposited at 400 °C was about 150 nm (Fig. 2b). Bigger coverages are required to completely cover the surface by ridges at 500 °C.

**Fig. 3:**
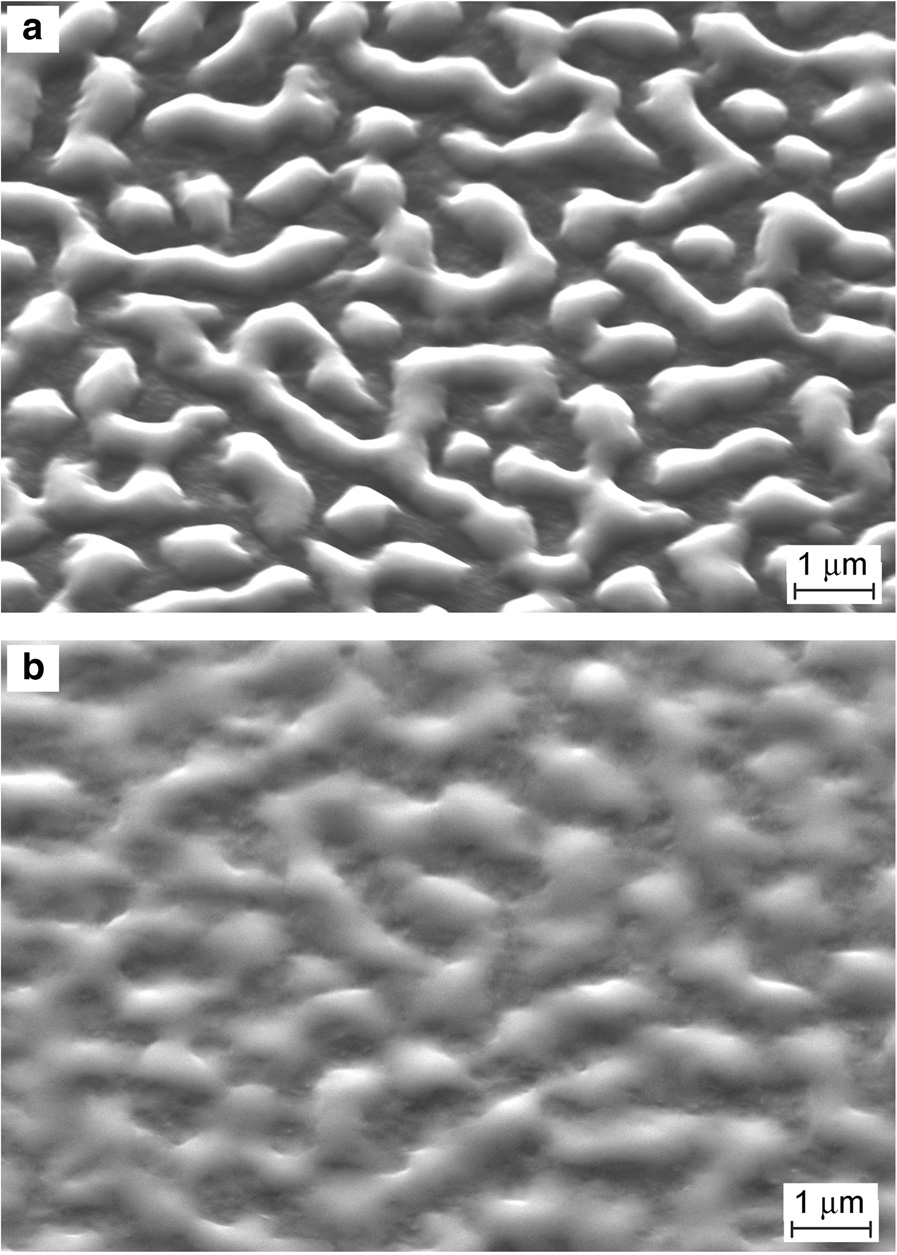
SEM images of the surfaces prepared by 30 Ge depositions on Si(100) at 400 °C and then annealed **a** at 800 °C for 30 min and **b** at 1000 °C for 15 min

Ge layers grown on Si(100) at 400 °C are thermally unstable due to the lattice strain and large surface and interface energies. Annealing at 800 °C of the samples covered with 30 nm Ge led to the smoothing of islands and ridges (Fig. 3a). The whole surface became smoothed after annealing at the higher temperature of 1000 °C (Fig. 3b). However, the annealing at 1000 °C for 60 min did not provide obtaining an atomically flat surface despite the possible Ge sublimation.

**Fig. 4:**
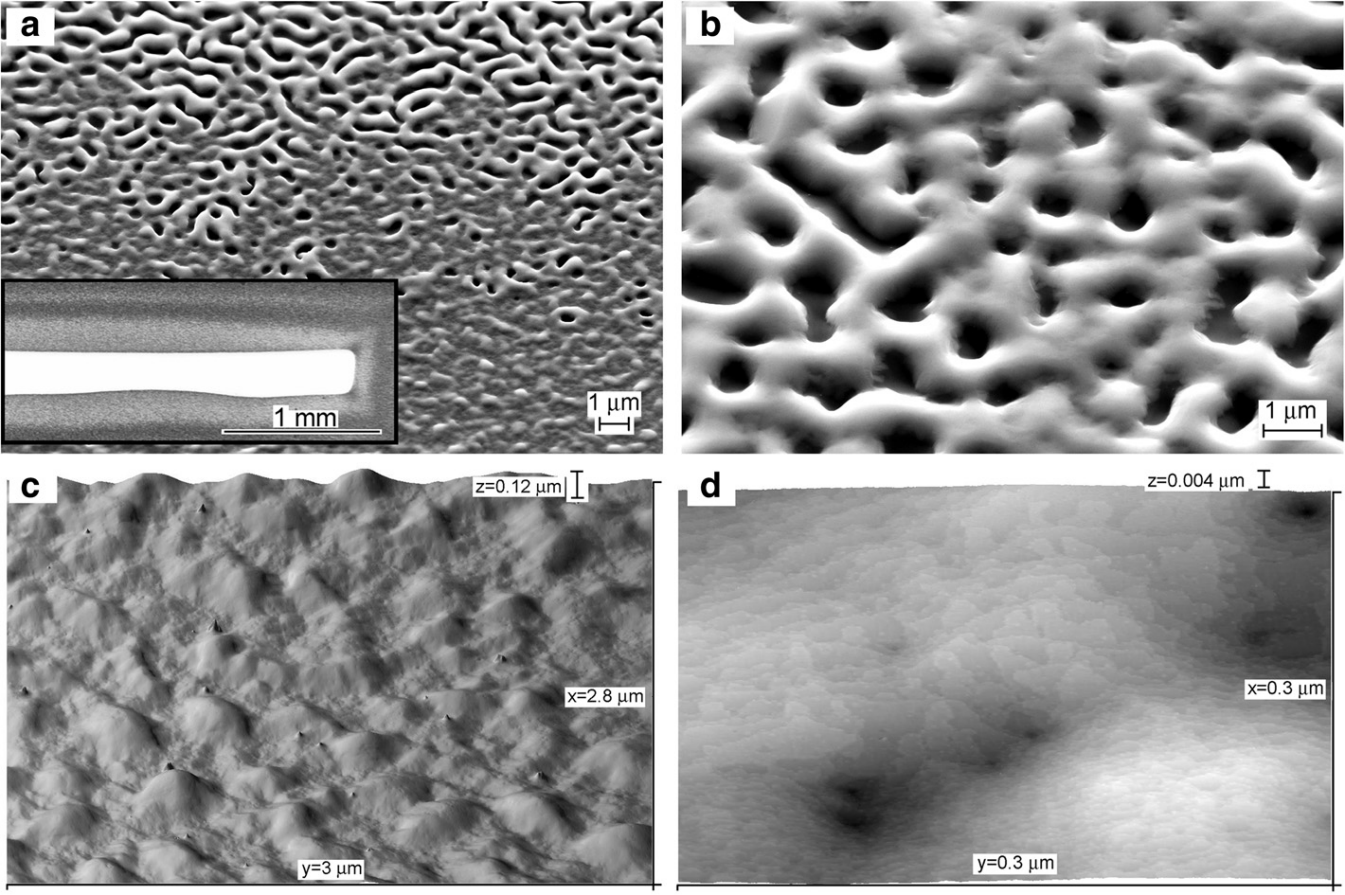
**a**, **b** SEM and **c**, **d** STM images of sample surfaces after 60 nm Ge deposition on Si(100) at 400 °C and subsequent annealing at 850 °C for 60 min. The *inset* in (**a**) shows the photo of the sample with the bright area in the center, representing the continuous SiGe layer which looks like a mirror, and the *gray area* around, represents the porous SiGe layer

The annealing of the samples with thicker Ge layers of about 60 nm at 800–900 °C resulted in the formation of two essentially different areas on the surface. The surface image with a boundary between them is shown in Fig. 4a. In the central part of the sample, the initial Ge ridges spread over the flat areas between them forming a continuous layer with a rough surface morphology (Fig. 4c, d). This sample area visually looks flat in the photo (inset in Fig. 4a). This was similar to that observed during annealing of the samples covered with 30 nm Ge (Fig. 3). However, around the central part of the sample surface, another surface morphology was formed. It is composed of open substrate surface areas surrounded by large ridges with smoothed edges (Fig. 4b) and it visually looks as a rough surface (inset in Fig. 4a). This sample surface area can be called a porous SiGe layer. The formation of the surface morphology similar to that of the porous layers was previously observed after the high-temperature annealing of Si(111) substrates covered with Ge [16].

Nevertheless, there are significant differences in the porous SiGe layer formation on Si(100) and Si(111). On Si(111), the radical transformation in the surface morphology occurred quickly under annealing at temperatures above 700 °C and for any deposited Ge coverage studied (up to 150 nm) [16]. Whereas, the porous SiGe layer formation on Si(100) began from the sample edges and the boundary between the continuous and porous layers moved slowly to the sample center with the rate of ~10 μm/min. The Ge coverage for which the porous layer formation was observed was also limited from about 50 to 100 nm.

In the surface area, where the porous SiGe layer is not formed under annealing, the continuous SiGe layer contains a high concentration of threading dislocations. The dislocations form small craters on the surface, as seen in the STM images (Fig. 4d). The number of the dislocations gradually decreases and the surface becomes smoother during long-time annealing at 1100 °C.

**Fig. 5:**
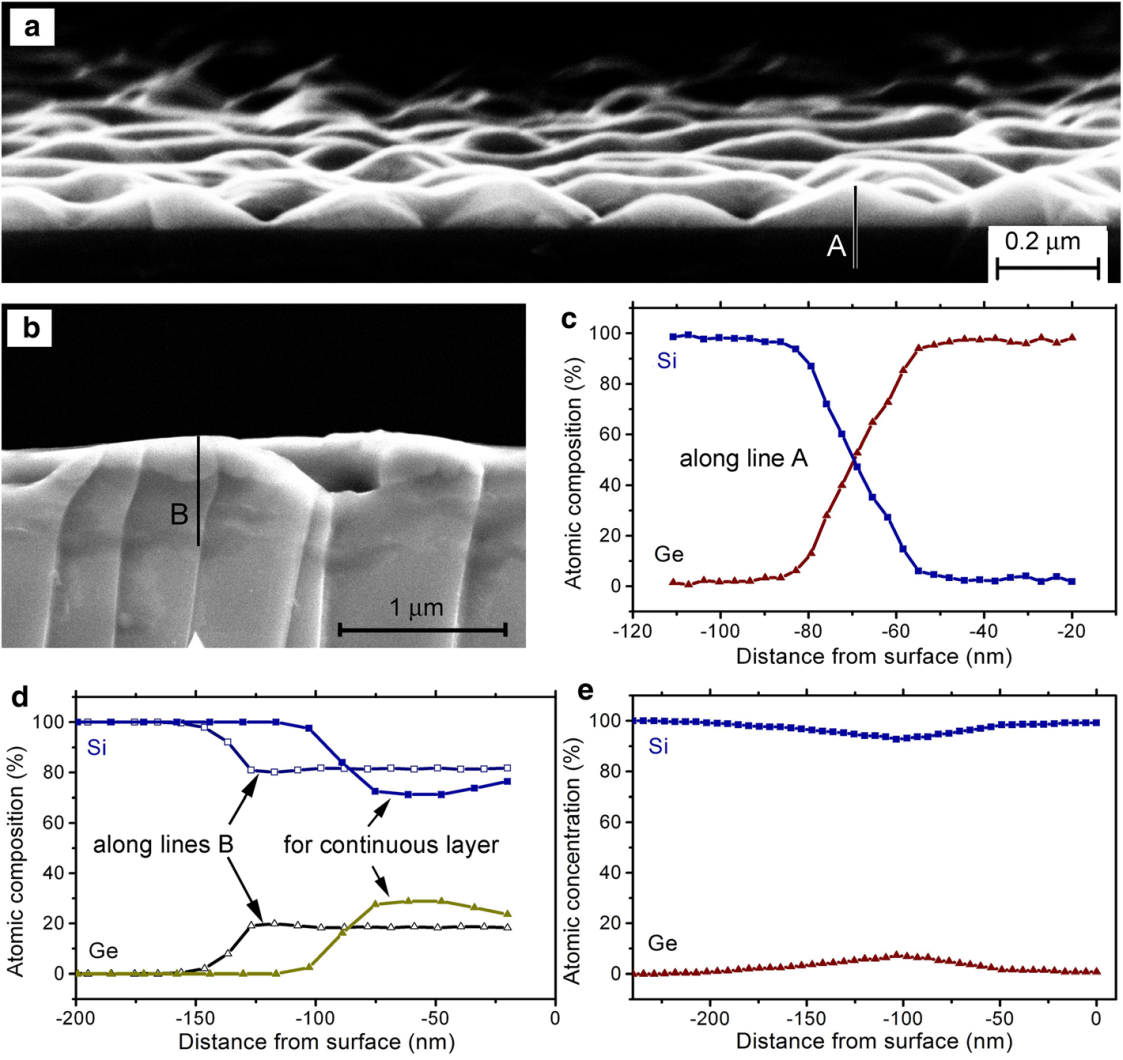
SEM images of cleaved samples prepared by the 60 nm Ge deposition on Si(100) **a** without post-growth annealing and **b** after the annealing at 850 °C for 60 min from the area of porous layers. **c**, **e** EDX data for the chemical composition. **c** Chemical composition obtained along line *A* in (**a**). **d** Comparison of the data obtained for the porous layer along line B in (**b**) and for a continuous layer, as marked in (**d**). **e** The data for the cleaved sample after the 150 nm Ge deposition on Si(100) with subsequent annealing at 1100 °C for 5 min

The high-temperature annealing initiates mass transport, which is accompanied by Si-Ge intermixing with Ge diffusion into the substrate. The chemical composition of the surface layers was obtained by EDX using sample cleavages. The SEM images of cleavages of samples prepared by the 60 nm Ge deposition on Si(100) without post-growth annealing and with annealing at 850 °C, which leads to the formation of porous layers, are shown in Fig. 5a, b, respectively. The layers grown at 400 °C (without subsequent annealing) are composed of Ge and have a sharp interface with the Si substrate. However, EDX data represent the chemical composition distribution across the Si/Ge interface as not sharp, but broadened for about 25 nm within which about 90 % changes in the chemical composition took place (Fig. 5c). This value gives the spatial resolution parameter of the EDX method for the incident e-beam energy of 3 keV used in our measurements.

Obtained EDX data for the different areas of the same sample, i.e., for areas of porous and continuous SiGe layers exhibit an essential difference from the viewpoint of spatial distribution of their chemical composition (Fig. 5 d). The SiGe spatial distribution in the continuous layer demonstrates that it is the result of gradual decay of the deposited Ge ridges by means of Si-Ge intermixing under the annealing at 850 °C. This occurs by the interdiffusion via the interface between the deposited Ge ridges and the substrate. In contrast, ridges of the porous layer have a uniform chemical composition of about Si_0.8_Ge_0.2_. The ridges/substrate interface width was about 25 nm, i.e., the same as the spatial resolution of our EDX measurements. This indicates that the interface between the porous SiGe layer and the substrate is sharp. Such distribution of SiGe composition can be the result of facilitated Si-Ge intermixing and diffusion within the surface layer, which may occur due to the surface layer melting with subsequent solidification.

The sample annealing at 1100 °C causes the Ge removal from surface layers (Fig 5e). The presence of small Ge amounts was observed in a broad region located at a depth of about 100 nm from the surface. This indicates that the Ge sublimation is the process additional to the Ge diffusion into the substrate, which produces a significant contribution into the spatial SiGe distribution in the surface layer.

The comparison of high-temperature behavior of Ge layers deposited on different Si substrates shows that the porous SiGe layer formation occurs on Si(111) easier than on Si(100). This depends on the relation between the rates of processes in surface layers. As suggested here, the porous SiGe layer formation is the result of surface melting which is significantly facilitated by the strong stain caused by the Si-Ge lattice mismatch. It can also be suggested that the same reason is responsible for the porous SiGe layer formation on Si(111) [16, 22]. Under annealing at temperatures in the range from 700 to 900 °C, two processes compete with each other. These are intermixing of Si and Ge atoms and their diffusion along the surface from one side and Ge diffusion into the Si substrate from the other side. Since Si(111) is the close-packed atomic plane, the diffusion along the Si(111) plane occurs easier than across it. Whereas, the diffusion condition along the Si(100) plane and across it is about the same. This difference in the diffusion conditions can be the reason for the different behavior of Ge layers on Si(111) and Si(100) surfaces. The better conditions for Ge atom diffusion into the Si substrate cause faster Si-Ge intermixing and, hence, faster stain relaxation under the annealing in the case of Si(100). Due to strain weakening the conditions for the surface layer melting gradually disappear and may not provide its realization along the whole sample surface. This explains the observed coexistence of the porous and continuous SiGe layers on Si(100).

Surface morphology transitions under annealing in the conditions of dewetting provide the benefit in the total energy owing to the strain relaxation, surface and interface energy minimization, intermixing, and structural defect optimization. Dewetting is widely used to produce the formation of very different surface morphologies [23–25]. It can occur through solid-state dewetting or melting of surface layers. At the same time, surface layers can be melted partially only in the local areas where the lattice strain is the strongest [26]. In the case of Ge on Si, the strongest strain appears along perimeters of Ge islands or ridges, i.e., at their edges [16, 27]. However, Ge layer melting on Si(100) in such local areas with subsequent Si-Ge intermixing is not sufficient for the formation of porous SiGe layers. It is important that they are formed as a result of dewetting during solidification. The possibility of preferable melting of local surface areas explains the fact that the porous layers are not formed when the Ge amount deposited on Si(100) exceeds 100 nm. So thick Ge layers almost do not contain open windows of the Si substrate and, hence, they do not contain local areas with strong lattice strain at edges of islands or ridges.

In the case of Ge layers on Si(111), it was shown that the porous layer formation, i.e., the layers which contain open windows of the Si substrate, provides the significant benefit in the total energy due to the reduction of SiGe/Si interface energy [16]. The energy benefit is proportional to the portion of the surface which is free from SiGe. It could be mentioned that the porous layer formation in these cases occurs despite the increase in the total surface energy due to formation of tall ridges.

There is another process which along with Si-Ge intermixing reduces the lattice strain under high-temperature annealing. This is the introduction of threading dislocations which are formed with a high concentration in the surface layers. Dislocations produce deep energy levels in the Si and SiGe bandgaps [28, 29]. The appearance of the deep levels creates the conditions for the luminescence in the near infrared spectral range [30, 31] in which the Si substrates are transparent. This circumstance makes the dislocation-rich SiGe layers whose formation described here to be interesting with respect to the study of their light absorption and luminescence properties.

The surface morphologies obtained under the high-temperature annealing of the samples with Ge layers deposited on Si(100) at the relatively low temperatures is drastically different from those formed by Ge deposition on Si(100) directly at high temperatures. In the latter case, the formation of individual island arrays takes place. The islands exhibit the tendency to ordering when they grow in the condition close to dynamic equilibrium between the islands growth and their decay due to Si-Ge intermixing with Ge diffusion into the Si substrate [9]. However, the ordered island or pore formation does not occur during high-temperature annealing as it was predicted by the simulation of the layer evolution [32]. The calculations did not account for the Si-Ge intermixing and the possible Si surface dewetting which take place in the Si-Ge system at high temperatures [22, 24].

## Conclusions

Ge layers deposited on Si(100) at relatively low temperatures (400–500 °C) are composed of separated large dome-like islands or ridges at higher Ge coverages. They are thermally unstable and transform into two surface areas with different morphologies depending on high-temperature annealing conditions and on the amount of deposited Ge. One of the areas is a porous SiGe layer containing open windows of the Si substrate, which is similar to that previously observed for Ge layers on Si(111). Their formation is associated with strain-induced surface melting followed by subsequent solidification in the conditions of SiGe dewetting on Si. The other surface area is a continuous SiGe layer with a rough surface morphology, which is preferably formed when the amount of deposited Ge was less than 50 or more than 100 nm. It is suggested that the conditions for the formation of porous or continuous SiGe areas are determined by the competition between processes such as intermixing of Si and Ge atoms with their diffusion along the surface and Ge diffusion into the Si substrate. In local surface areas where the lattice strain is sufficiently strong, the surface layer melting takes place that significantly facilitates Si-Ge intermixing with surface diffusion leading to the porous SiGe layer formation. However, if the melting does not occur, the deposited Ge islands or ridges gradually transforms into the continuous SiGe layer by means of their decay along the surface and Ge diffusion into the substrate. It is discussed that easier porous SiGe layer formation on Si(111) than on Si(100) originates from the different conditions for the Ge surface and bulk diffusion on Si substrates with such surface orientations.

## Abbreviations

EDX: Energy-dispersive X-ray spectroscopy
SEM: Scanning electron microscopy
STM: Scanning tunneling microscopy

## References

[1] Michel J, Liu J, Kimerling LC (2010). High-performance Ge-on-Si photodetectors. Nat Photon.

[2] Süess MJ, Geiger R, Minamisawa RA, Schiefler G, Frigerio J, Chrastina D, Isella G, Spolenak R, Faist J, Sigg H (2013). Analysis of enhanced light emission from highly strained germanium microbridges. Nat Phot.

[3] Chakraborty PS, Cardoso AS, Wier BR, Omprakash AP, Cressler JD, Kaynak M, Tillack B (2014). A 0.8 THz SiGe HBT operating at 4.3K. IEEE Elec Dev Lett.

[4] Mo YW, Savage DE, Swarzentruber BS, Lagally MG (1990). Kinetic pathway in Stranski-Krastanov growth of Ge on Si(001). Phys Rev Lett.

[5] Shklyaev AA, Ichikawa M (2008). Extremely dense arrays of germanium and silicon nanostructures. Phys-Usp.

[6] Baskaran A, Smereka P (2012). Mechanisms of Stranski-Krastanov growth. J Appl Phys.

[7] Dashiell MW, Denker U, Müller C, Costantini G, Manzano C, Kern K, Schmidt OG (2002). Photoluminescence of ultrasmall Ge quantum dots grown by molecular-beam epitaxy at low temperatures. Appl Phys Lett.

[8] Talochkin AB, Chistokhin IB, Mashanov VI (2016). Photoconductivity of ultra-thin Ge (GeSn) layers grown in Si by low-temperature molecular beam epitaxy. J Appl Phys.

[9] Shklyaev AA, Budazhapova AE (2016). Ge deposition on Si(100) in the conditions close to dynamic equilibrium between islands growth and their decay. Appl Surf Sci.

[10] Chaparro SA, Zhang Y, Drucker J (2000). Strain relief via trench formation in Ge/Si (100) islands. Appl Phys Lett.

[11] Talochkin AB, Shklyaev AA, Mashanov VI (2014). Super-dense array of Ge quantum dots grown on Si (100) by low-temperature molecular beam epitaxy. J Appl Phys.

[12] Nakajima K, Konishi A, Kimura K (1999). Direct observation of intermixing at Ge/Si (001) interfaces by high-resolution Rutherford backscattering spectroscopy. Phys Rev Lett.

[13] Capellini G, De Seta M, Evangelisti F (2001). SiGe intermixing in Ge/Si(100) islands. Appl Phys Lett.

[14] Baranov AV, Fedorov AV, Perova TS, Moore RA, Yam V, Bouchier D, Le Thanh V, Berwick K (2006). Analysis of strain and intermixing in single-layer Ge∕Si quantum dots using polarized Raman spectroscopy. Phys Rev B.

[15] Shklyaev AA, Romanyuk KN, Kosolobov SS (2014). Surface morphology of Ge layers epitaxially grown on bare and oxidized Si(001) and Si(111) substrates. Surf Sci.

[16] Shklyaev AA, Ponomarev KE (2015). Strain-induced Ge segregation on Si at high temperatures. J Cryst Growth.

[17] Katsaros G, Costantini G, Stoffel M, Esteban R, Bittner AM, Rastelli A, Denker U, Schmidt OG, Kern K (2005). Kinetic origin of island intermixing during the growth of Ge on Si (001). Phys Rev B.

[18] Richard MI, Malachias A, Stoffel M, Merdzhanova T, Schmidt OG, Renaud G, Metzger TH, Schülli TU (2016). Temperature evolution of defects and atomic ordering in Si_1− x_Ge_x_ islands on Si(001). J Appl Phys.

[19] Medeiros-Ribeiro G, Bratkovski AM, Kamins TI, Ohlberg DAA, Williams RS (1998). Shape transition of germanium nanocrystals on a silicon (001) surface from pyramids to domes. Science.

[20] Shklyaev AA, Shibata M, Ichikawa M (1998). Ge islands on Si(111) at coverages near the transition from two-dimensional to three-dimensional growth. Surf Sci.

[21] Liao XZ, Zou J, Cockayne DJH, Qin J, Jiang ZM, Wang X, Leon R (1999). Strain relaxation by alloying effects in Ge islands grown on Si(001). Phys Rev B.

[22] Shklyaev A, Bolotov L, Poborchii V, Tada T (2015). Properties of three-dimensional structures prepared by Ge dewetting from Si(111) at high temperatures. J Appl Phys.

[23] Thompson CV (2012). Solid-state dewetting of thin films. Annu Rev Matter Res.

[24] Leroy F, Cheynis F, Almadori Y, Curiotto S, Trautmann M, Barbe JC, Müller P (2016). How to control solid state dewetting: a short review. Surf Sci Rep.

[25] Naffouti M, David T, Benkouider A, Favre L, Ronda A, Berbezier I, Bidault S, Bonod N, Abbarchi M (2016). Fabrication of poly-crystalline Si-based Mie resonators via amorphous Si on SiO_2_ dewetting. Nanoscale.

[26] Tartaglino U, Tosatti E (2003). Strain effects at solid surfaces near the melting point. Surf Sci.

[27] Gatti R, Marzegalli A, Zinovyev VA, Montalenti F, Miglio L (2008). Modeling the plastic relaxation onset in realistic SiGe islands on Si(001). Phys Rev B.

[28] Drozdov NA, Patrin AA, Tkachev VD (1976). Recombination radiation on dislocations in silicon. Jetp Lett.

[29] Shklyaev AA, Vdovin VI, Volodin VA, Gulyaev DV, Kozhukhov AS, Sakuraba M, Murota J (2015). Structure and optical properties of Si and SiGe layers grown on SiO_2_ by chemical vapor deposition. Thin Solid Films.

[30] Shklyaev AA, Dultsev FN, Mogilnikov KP, Latyshev AV, Ichikawa M (2010). Electroluminescence of dislocation-rich Si layers grown using oxidized Si surfaces. J Phys D Appl Phys.

[31] Lausch D, Mehl T, Petter K, Flø AS, Burud I, Olsen E (2016). Classification of crystal defects in multicrystalline silicon solar cells and wafer using spectrally and spatially resolved photoluminescence. J Appl Phys.

[32] Pang Y, Huang R (2009). Effect of elastic anisotropy on surface pattern evolution of epitaxial thin films. Int J Solids and Structures.

